# CALENDAR

**DOI:** 10.1054/bjoc.2001.1978

**Published:** 2001-07


					26-28 September 2001
8th Hong Kong International Cancer Congress
Hong Kong Academy of Medicine Building, Hong Kong
Further information from:
8th HKICC Congress Secretariat, Department of Surgery,
University of Hong Kong Medical Centre, Queen Mary Hospital,
Hong Kong. Tel: + 852 2818 0232/2855 4235;
Fax: + 852 2818 1186; E-mail: mededcon@hku.hk
4-6 October 2001
8th International Conference on Gastrointestinal
Oncology: Gastric and Esophageal Cancer
Washington DC, USA
Jointly sponsored by The George Washington University,
Institut Bergonie and I' Universite Victor Segalen (University
of Bordeaux II).
Further information from:
Office of Continuing Medical Education, The George Washington
University Medical Center, 2300 K Street NW, Washington DC
20037, USA. Tel: +1 202 994 4285; Fax: +1 202 994 1791
1-4 November 2001
13th Biennial Congress of Asian Surgical Association
Shangrila Hotel, Singapore
Further information from:
Congress Secretariat, 13th Biennial Congress of Asian Surgical
Association, c/o Academy of Medicine, Singapore, 142 Neil
Road, Singapore 088871. Tel: + 65 223 8968; Fax: + 65 225
5155; E-mail: main-ac@academyofmedicine.edu.sg;
Website: http://www.academy of medicine.edu.sg
CALENDAR
British Journal of Cancer (2001) 85(2), 312
(c) 2001 Cancer Research Campaign
doi: 10.1054/ bjoc.2001.1978, available online at http://www.idealibrary.com on
312

				

